# Supplementary material for: Oxygen desaturation and lung ultrasonography as markers of diffuse parenchymal lung diseases severity
Source: PLoS One. 2025 May 9;20(5):e0322657. doi: 10.1371/journal.pone.0322657 (PMC12063835; doi:10.1371/journal.pone.0322657)
Supplement: S1 File — (DOCX) [file pone.0322657.s001.docx]

**S1 Table. The baseline characteristics of the participants (DPLD and control groups)**

| **Variable** | **Patients (n=31)** | **Controls (n=20)** | **p value** |
| --- | --- | --- | --- |
| **Age (years)** | 44.7 ± 11.4 | 44.9 ± 10.5 | 0.959 |
| **Gender**  **Male / Female** | 6 (19.4) / 25 (80.6) | 8 (40.0) / 12 (60.0) | 0.196 |
| **BMI** | 25.94 ± 3.87 | 26.36 ± 2.38 | 0.624 |
| **Smoking history**  **Smoker / non-smoker** | 3 (9.7) / 28 (90.3) | 0 (0) / 20 (100) | 0.270 |
| **Spirometry**  **FVC (L)**  **FVC %Pred**  **FVC< 50%**  **FEV_1_ (L)**  **FEV_1_ %Pred**  **FEV_1_ /FVC** | 1.97 (1.34 – 2.52)  57.87 ± 25.36  12 (38.7)  1.67 (1.06 – 1.89)  63.0 (40.5 – 76.5)  81.71 (71.85 – 84.39) | 3.27 (2.91 – 1.04)  90.50 ± 7.89  0 (0)  3.07 (2.69 – 3.67)  88.5 (84.8 - 94.0)  92.30 (86.07 – 96.15) | <0.001*  0.001*  <0.001*  <0.001*  <0.001* |
| **mPAP** | 27.5 ± 12.86 | 18.44 ± 3.75 | 0.002* |
| **6MWT**  **6MWD (meters)**  **6MWT nadir SpO_2_ (%)** | 270.0 (230.0 – 313.0)  95.0 (91.0 – 97.0) | 410.0 (390.0 – 420.0)  98.0 (98.0 – 99.0) | <0.001*  <0.001* |
| **HRCT**  **Warrick score** | 16.0 ± 7 | 0.0 | <0.001* |
| **LUS**  **Total n. of B-lines** | 41.0 (28.0 – 68.5) | 0.0 (0.0 – 1.0) | <0.001* |
| **Polygraph**  **ODI (events/ h)**  **T90 (% of sleep)**  **AHI (events/ h)**  **Nocturnal nadir SpO_2_ (%)** | 3.66 ± 3.82  13.20 ± 21.58  0.81 ± 1.85  80.63 ± 8.96 | 0.96 ± 0.89  0.05 ± 0.22  0.020 ± 0.06  91.60 ± 2.25 | 0.006*  <0.001*  <0.001*  0.009* |

Abbreviations; n: number, BMI: body mass index, mPAP: mean pulmonary artery pressure, FVC: forced vital capacity, L: liter, FEV_1_: forced expired volume in 1 second, FEF_25-75_: forced expiratory flow between 25 to 75 percentile, SpO_2_: oxygen saturation, 6MWD: 6-minute walk distance, 6MWT: 6-minute walk test, ODI: oxygen desaturation index, AHI: apnea / hypopnea index, h: hour, T90: timed oxygen saturation < 90%. Qualitative data are presented as number (%) and quantitative data are presented as median (IQR) or mean ± SD according to data distribution. * Significant p value < 0.05.

**S2 Table. Correlations between LUS parameters and various physiological / HRCT parameters**

|  | | **B-lines (≥3)** | **Total N. of B-lines** | **Limited sliding** | **Pleural fragmentation** | **Pleural irregularity** | **Pleural thickness** | **Subpleural nodules** |
| --- | --- | --- | --- | --- | --- | --- | --- | --- |
| **FVC % pred.** | ***r*** | -0.753^*^ | -0.693^*^ | -0.354^*^ | -0.700^*^ | -0.722^*^ | -0.352^*^ | -0.372^*^ |
|  | ***p*** | <0.001 | <0.001 | 0.011 | <0.001 | <0.001 | 0.011 | 0.007 |
| **FVC (L)** | ***r*** | -0.655^*^ | -0.599^*^ | -0.267 | -0.555^*^ | -0.666^*^ | -0.319^*^ | -0.377^*^ |
|  | ***p*** | <0.001 | <0.001 | 0.058 | <0.001 | <0.001 | 0.023 | 0.006 |
| **FEV_1_% pred.** | ***r*** | -0.725^*^ | -0.687^*^ | -0.347^*^ | -0.624^*^ | -0.742^*^ | -0.357^*^ | -0.447^*^ |
|  | ***p*** | <0.001 | <0.001 | 0.013 | <0.001 | <0.001 | 0.01 | 0.001 |
| **Baseline SpO_2_** | ***r*** | -0.589^*^ | -0.552^*^ | -0.359^*^ | -0.385^*^ | -0.537^*^ | -0.290^*^ | -0.137 |
|  | ***p*** | <0.001 | <0.001 | 0.01 | 0.005 | <0.001 | 0.039 | 0.338 |
| **6MWT nadir SpO_2_** | ***r*** | -0.728^*^ | -0.732^*^ | -0.344^*^ | -0.558^*^ | -0.726^*^ | -0.446^*^ | -0.334^*^ |
|  | ***p*** | <0.001 | <0.001 | 0.018 | <0.001 | <0.001 | 0.002 | 0.022 |
| **T90** | ***r*** | 0.665^*^ | 0.629^*^ | 0.121 | 0.490^*^ | 0.674^*^ | 0.390^*^ | 0.413^*^ |
|  | ***p*** | <0.001 | <0.001 | 0.401 | <0.001 | <0.001 | 0.005 | 0.003 |
| **Nocturnal nadir SpO_2_** | ***r*** | -0.561^*^ | -0.581^*^ | -0.180 | -0.411^*^ | -0.657^*^ | -0.352^*^ | -0.455^*^ |
|  | ***p*** | <0.001 | <0.001 | 0.212 | 0.003 | <0.001 | 0.012 | 0.001 |
| **MPAP** | ***r*** | 0.183 | 0.167 | -0.218 | 0.027 | 0.273 | 0.239 | 0.400^*^ |
|  | ***p*** | 0.286 | 0.329 | 0.202 | 0.875 | 0.107 | 0.161 | 0.016 |
| **Warrick Score** | ***r*** | 0.874^*^ | 0.875^*^ | 0.316^*^ | 0.814^*^ | 0.895^*^ | 0.428^*^ | 0.468^**^ |
|  | ***p*** | <0.001 | <0.001 | 0.024 | <0.001 | <0.001 | 0.002 | 0.001 |

**S3 Table. Correlations between the various physiological parameters and Warrick score**

|  | | **FVC % pred.** | **FVC (L)** | **FEV_1_ % pred.** | **Basal SpO_2_** | **6MWT nadir SpO_2_** | **T90** | **Nocturnal nadir SpO_2_** | **MPAP** | **Warrick score** |
| --- | --- | --- | --- | --- | --- | --- | --- | --- | --- | --- |
| **Age** | ***r*** | -0.017 | -0.247 | -0.027 | -0.144 | -0.149 | 0.020 | -0.028 | 0.324 | 0.034 |
|  | ***p*** | 0.904 | 0.081 | 0.853 | 0.312 | 0.317 | 0.892 | 0.845 | 0.054 | 0.812 |
| **FVC % pred.** | ***r*** | NA | 0.842^*^ | 0.930^*^ | 0.589^*^ | 0.683^*^ | -0.562^*^ | 0.537^*^ | -0.052 | -0.815^*^ |
|  | ***p*** | NA | <0.001 | <0.001 | <0.001 | <0.001 | <0.001 | <0.001 | 0.765 | <0.001 |
| **FVC (L)** | ***r*** | 0.842^*^ | NA | 0.833^*^ | 0.568^*^ | 0.684^*^ | -0.613^*^ | 0.598^*^ | -0.204 | -0.753^*^ |
|  | ***p*** | <0.001 | NA | <0.001 | <0.001 | <0.001 | <0.001 | <0.001 | 0.232 | <0.001 |
| **FEV_1_ % pred.** | ***r*** | 0.930^*^ | 0.833^*^ | NA | 0.591^*^ | 0.728^*^ | -0.584^*^ | 0.594^*^ | -0.119 | -0.815^*^ |
|  | ***p*** | <0.001 | <0.001 | NA | <0.001 | <0.001 | <0.001 | <0.001 | 0.488 | <0.001 |
| **Basal SpO_2_** | ***r*** | 0.589^*^ | 0.568^*^ | 0.591^*^ | NA | 0.781^*^ | -0.719^*^ | 0.590^*^ | -0.272 | -0.616^*^ |
|  | ***p*** | <0.001 | <0.001 | <0.001 | NA | <0.001 | <0.001 | <0.001 | 0.109 | <0.001 |
| **6MWT nadir SpO_2_** | ***r*** | 0.683^*^ | 0.684^*^ | 0.728^*^ | 0.781^*^ | NA | -0.746^*^ | 0.669^*^ | -0.245 | -0.833^*^ |
|  | ***p*** | <0.001 | <0.001 | <0.001 | <0.001 | NA | <0.001 | <0.001 | 0.177 | <0.001 |
| **T90** | ***r*** | -0.562^*^ | -0.613^*^ | -0.584^*^ | -0.719^*^ | -0.746^*^ | NA | -0.842^*^ | 0.473^*^ | 0.697^*^ |
|  | ***p*** | <0.001 | <0.001 | <0.001 | <0.001 | <0.001 | NA | <0.001 | 0.004 | <0.001 |
| **Nocturnal nadir SpO_2_** | ***r*** | 0.537^*^ | 0.598^*^ | 0.594^*^ | 0.590^*^ | 0.669^*^ | -0.842^*^ | NA | -0.531^*^ | -0.685^*^ |
|  | ***p*** | <0.001 | <0.001 | <0.001 | <0.001 | <0.001 | <0.001 | NA | 0.001 | <0.001 |

NA: not assessed

**S4 Table. Univariate logistic regression analysis regarding the severity of FVC (as ≤ 50% considered severe disease)**

|  | **B** | **S.E.** | **Sig.** | **OR** | **95%CI for OR** | |
| --- | --- | --- | --- | --- | --- | --- |
|  |  |  |  |  | **Lower** | **Upper** |
| **Age** | -0.504 | 0.396 | 0.203 | 0.604 | 0.278 | 1.313 |
| **Gender** | 0.799 | 0.849 | 0.347 | 2.222 | 0.421 | 11.728 |
| **Severity of PAH** | 0.200 | 0.320 | 0.532 | 1.221 | 0.652 | 2.287 |
| **6-MWT nadir SpO_2_** | 1.147 | 0.348 | 0.001* | 3.149 | 1.593 | 6.223 |
| **T90** | 0.394 | 0.205 | 0.055 | 1.483 | 0.992 | 2.218 |
| **Nadir nocturnal SpO_2_** | 0.882 | 0.322 | 0.006* | 2.415 | 1.284 | 4.540 |
| **Zones with B-lines ≥ 3** | 0.983 | 0.329 | 0.003* | 2.673 | 1.403 | 5.092 |
| **US pleural abnormalities** | 3.091 | 1.098 | 0.005* | 22.00 | 2.556 | 189.369 |
| **Warrick Score** | 3.066 | 1.123 | 0.006* | 21.449 | 2.376 | 193.653 |
| **US Score** | 1.636 | 0.576 | 0.004* | *5.135* | *1.662* | *15.870* |
| **Severity Score** | 0.895 | 0.289 | 0.002* | 2.448 | 1.390 | 4.311 |

LUS Score: the sum of B-lines ≥ 3 and pleural abnormalities (either irregularity and fragmentation); Severity Score: US score, 6-MWT Nadir SaO_2,_ T90
