# Supplementary figures and images for: Oxygen desaturation and lung ultrasonography as markers of diffuse parenchymal lung diseases severity
Source: PLoS One. 2025 May 9;20(5):e0322657. doi: 10.1371/journal.pone.0322657 (PMC12063835; doi:10.1371/journal.pone.0322657)

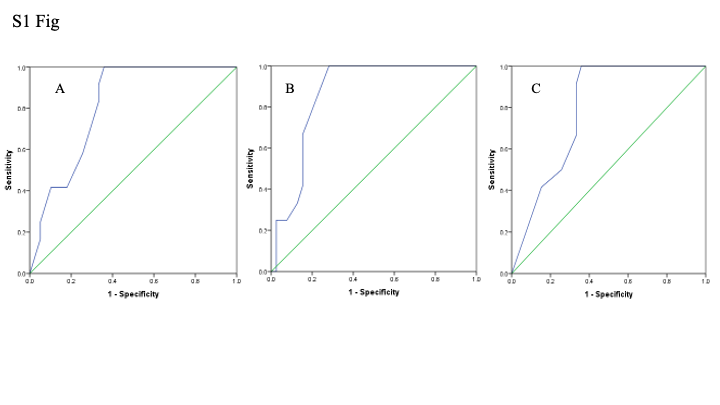

Supplement: S1 Fig — Number of zones with B-lines ≥ 3 (AUC = 0.812, CI 95% = 0.696–0.928, p = 0.001), B. Number of zones with pleural fragmentation (AUC = 0.862, CI 95% = 0.764–0.961, p < 0.001), C. Number of zones with pleural irregularity (AUC = 0.790, CI 95% = 0.668–0.911, p = 0.003). (TIF) [file pone.0322657.s002.tif]

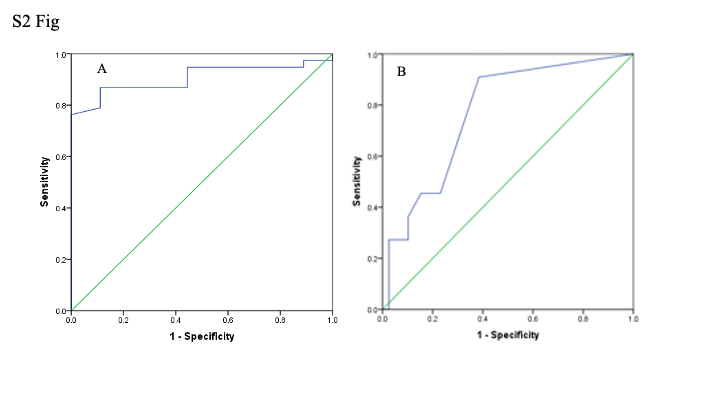

Supplement: S2 Fig — (A. AUC = 0.905, CI 95% = 0.819–0.991, p < 0.001) and T90 (B. AUC = 0.769, CI 95% = 0.622–0.917, p = 0.007) in predicting severe fibrotic DPLD (i.e., those with evidence of HRCT fibrosis and FVC ≤ 50%). (TIF) [file pone.0322657.s003.tif]
