# Supplementary material for: Oxygen desaturation and lung ultrasonography as markers of diffuse parenchymal lung diseases severity
Source: PLoS One. 2025 May 9;20(5):e0322657. doi: 10.1371/journal.pone.0322657 (PMC12063835; doi:10.1371/journal.pone.0322657)
Supplement: Attachment — Submitted filename: response to reviewers 2.docx [file pone.0322657.s006.docx]

Dear Prof. Dr. Vincenzo Lionetti, MD, PhD
Academic Editor

I would like to thank the editor in-chief, the academic editor, the editorial office, and the reviewers for your time and effort in reviewing the paper and the valuable comments. We truly think that the manuscript has improved sensibly after your suggestions. We would like to respond all reviewer’s comments point by point and fulfil all the journal requirements.

**Journal Requirements:**

Please review your reference list to ensure that it is complete and correct. If you have cited papers that have been retracted, please include the rationale for doing so in the manuscript text, or remove these references and replace them with relevant current references. Any changes to the reference list should be mentioned in the rebuttal letter that accompanies your revised manuscript. If you need to cite a retracted article, indicate the article’s retracted status in the References list and also include a citation and full reference for the retraction notice.

***Response****.* We reviewed the reference lists and we corrected them accordingly (marked in the references section). In the revised version of manuscript, we have not cited papers that have been retracted. All the retracted references in the 1^st^ revised manuscript were following the reviewers’ comments to revise the introduction mainly and to less extent the discussion to focus more directly on the study's relevance without excessive background information; so, we removed references related to modified sections (references 2, 7, 15, 29, 33, 34 in the old version) and add others to support the rationale of the study (references 2, 3, 5 in the revised version; the introduction section). Additionally, the newly added references were to support the parts added to the study following the reviewers’ comment like in the statistics, discussion and limitations of study (references 25, 30, 31, 35, 36, 37 in the revised version; the discussion/limitations section). As being requested, we mentioned the changes in the reference list in the rebuttal letter that accompanies the final revised manuscript (shown below).

*The Retracted References:* (in the old version)

2.Warrick JH, et al. High resolution computed tomography in early scleroderma lung disease. *J Rheumatol.* 1991;18(10):1520-1528;

7. Troy LK, et al. Nocturnal hypoxaemia is associated with adverse outcomes in interstitial lung disease. *Respirology.* 2019;24(10):996-1004;

15. Johns MW. A new method for measuring daytime sleepiness: the Epworth sleepiness scale. *Sleep.* 1991;14(6):540-545;

29. Bhat I, et al. Role of high-resolution computed tomography chest in interstitial lung diseases. *Int J Sci Stud.* 2016;4:20-26;

33. Caminati A, et al. Walking distance on 6-MWT is a prognostic factor in idiopathic pulmonary fibrosis. *Respir Med.* 2009;103(1):117-123;

34. Harari S, et al. The 6-min walk test as a primary end-point in interstitial lung disease. *Eur Respir Rev.* 2022;31(165).

*The newly included references*: (in the revised version)

2. Khor YH, et al. Exertional Desaturation and Prescription of Ambulatory Oxygen Therapy in Interstitial Lung Disease. Respir Care. 2019;64(3):299-306. Epub 2018/11/01. doi: 10.4187/respcare.06334.

3. Lama VN, et al. Prognostic value of desaturation during a 6-minute walk test in idiopathic interstitial pneumonia. Am J Respir Crit Care Med. 2003;168(9):1084-90. Epub 2003/08/15. doi: 10.1164/rccm.200302-219OC.

5. Kolilekas L, et al. Sleep oxygen desaturation predicts survival in idiopathic pulmonary fibrosis. J Clin Sleep Med. 2013;9(6):593-601. Epub 2013/06/19. doi: 10.5664/jcsm.2758.

25.	Chow S-C, Wang H, Shao J. Sample Size Calculations in Clinical Research (2nd ed.). Chapman and Hall/CRC. 2007. doi: https://doi.org/10.1201/9781584889830.

30.	Yasuda Y, et al. Analysis of nocturnal desaturation waveforms using algorithms in patients with idiopathic pulmonary fibrosis. Sleep Breath. 2022;26(3):1079-86. Epub 2021/08/23. doi: 10.1007/s11325-021-02456-3.

31.	Myall KJ, et al. Nocturnal Hypoxemia Associates With Symptom Progression and Mortality in Patients With Progressive Fibrotic Interstitial Lung Disease. Chest. 2023;164(5):1232-42. Epub 2023/05/16. doi: 10.1016/j.chest.2023.05.013.

35.	Laursen CB, et al. European Respiratory Society statement on thoracic ultrasound. Eur Respir J. 2021;57(3):2001519. doi: 10.1183/13993003.01519-2020.

36.	Elicker BM, et al. The role of high-resolution computed tomography in the follow-up of diffuse lung disease. Eur Respir Rev. 2017;26(144):170008. doi: 10.1183/16000617.0008-2017.

37.	Simon MA. Assessment and treatment of right ventricular failure. Nat Rev Cardiol. 2013;10(4):204-18. doi: 10.1038/nrcardio.2013.12.

**Reviewer #1:** I appreciate the effort in revising the paper, however thereare still some issues in the reporting of the results.

*Thank you for your valuable evaluation and your time and we would like to respond to your comments.*

- In the new Figure 3B the ROC curve of LUS score is not visible, moreover the authors state "Moreover, both LUS and severity predicting scores can significantly well differentiate between the DPLD and healthy control groups (AUC= 0.903, CI95%= 0.816 – 0.990, p < 0.001 for both LUS and severity scores, Fig 3B) as a score ≥ 2 had a sensitivity of 74.2% and 77.4% respectively with a specificity of 100% in predicting DPLD." This is not clear: do the severity score and LUS have the same AUC? then why the ROC of LUS is not reported in the figure?

***Response****.* We agree with the reviewer that this point carries some confusion. Yes, the severity score and LUS have the same AUC; so, the ROC of LUS is superimposed on that of the severity score resulting in one curve. The similarity of AUC for both scores in the figure 3B is expected as the comparison is carried out between the healthy population and those with DPLD. Accordingly, in order to present the ROC curve of both scores clearly, we separated the ROC of LUS and severity scores into 2 figures (shown below) to clarify this point. The modified figures are included in the manuscript as Fig 4A and 4B respectively (instead of Fig 3B “removed”) with correction of the text in the manuscript (lines 253-254) and the legend of figure 3 (lines 258, 263- 266).

- I strongly encourage the authors to revise the manuscript with the help of a native English speaker, as many sections are still difficult to read and appear verbose or convoluted.

***Response****.* Following the reviewer’s suggestion, a native English speaker reviewed the whole manuscript and grammatical mistakes / sentences were corrected (marked throughout the document).

**Reviewer #2:** The authors have adequately addressed the points raised and the overall quality of the manuscript is improved.

*Thank you for your valuable evaluation and your time.*
